# The Severity of Visceral Leishmaniasis Correlates with Elevated Levels of Serum IL-6, IL-27 and sCD14

**DOI:** 10.1371/journal.pntd.0004375

**Published:** 2016-01-27

**Authors:** Priscila L. dos Santos, Fabrícia A. de Oliveira, Micheli Luize B. Santos, Luana Celina S. Cunha, Michelle T. B. Lino, Michelle F. S. de Oliveira, Manuela O. M. Bomfim, Angela Maria Silva, Tatiana R. de Moura, Amélia R. de Jesus, Malcolm S. Duthie, Steven G. Reed, Roque P. de Almeida

**Affiliations:** 1 Laboratório de Biologia Molecular, Hospital Universitário, Universidade Federal de Sergipe, Aracaju, Brazil; 2 Programa de Pós Graduação em Ciências da Saúde, Universidade Federal de Sergipe, Aracaju, Brazil; 3 Instituto de Investigação em Imunologia, São Paulo, Brazil; 4 Infectious Diseases Research Institute (IDRI), Seattle, Washington, United States of America; Federal University of Minas Gerais, BRAZIL

## Abstract

**Background:**

Visceral leishmaniasis (VL) is a severe disease caused by infection with protozoa of the genus *Leishmania*. Classic VL is characterized by a systemic infection of phagocytic cells and an intense activation of the inflammatory response. It is unclear why 90% of infected individuals do not develop the disease while a minority develop the classical form. Furthermore, among those that develop disease, a small group progresses to more severe form that is unresponsive to treatment. The presence of inflammatory mediators in serum could theoretically help to control the infection. However, there is also a release of anti-inflammatory mediators that could interfere with the control of parasite multiplication. In this study, we took advantage of the spectrum of outcomes to test the hypothesis that the immune profile of individuals infected with *Leishmania (L*.*) infantum* is associated with the development and severity of disease.

**Methodology/Principal Findings:**

Sera from patients with confirmed diagnosis of VL were evaluated for the presence of numerous molecules, and levels compared with healthy control and asymptomatic infected individuals.

**Conclusions/Principal Findings:**

Although differences were not observed in LPS levels, higher levels of sCD14 were detected in VL patients. Our data suggest that *L*. *infantum* may activate the inflammatory response via CD14, stimulating a generalized inflammatory response with production of several cytokines and soluble molecules, including IFN-γ, IL-27, IL-10, IL-6 and sCD14. These molecules were strongly associated with hepatosplenomegaly, neutropenia and thrombocytopenia. We also observed that IL-6 levels greater than 200 pg/ml were strongly associated with death. Together our data reinforce the close relationship of IFN-γ, IL-10, IL-6, TNF-α and IL-27 in the immune dynamics of VL and suggest the direct participation of sCD14 in the activation of the immune response against *L*. *infantum*.

## Author Summary

Visceral leishmaniasis (VL) is a systemic, and the most severe, form of leishmaniasis. VL has high morbidity and leads to death if not properly treated. The interactions between the parasite and the host immune response lead to different clinical manifestations (either asymptomatic, oligosymptomatic or symptomatic). A comprehensive understanding of the immunological factors associated with VL outcome is critical for the development of immunotherapeutic or immunoprophylatic approaches to control this disease. In this study, we observed the correlation of IL-6, IL-27 and sCD14 levels with the pathogenesis of VL.

## Introduction

Visceral leishmaniasis (VL), also known as kala-azar, is a disease caused by infection with the intracellular protozoa *Leishmania (L*.*) donovani* (in the old world) and *L*. *infantum (chagasi) (*in the new world). Among tropical diseases, VL is ranked as fourth in morbidity and second in mortality, with 20,000 to 40,000 deaths per year [1,2].

Both *Leishmania donovani* and *L*. *infantum* multiply inside mononuclear phagocytes in the spleen, liver and bone marrow. About 90% of those subjects that are infected with *Leishmania* do not develop the classic symptoms of VL, and are considered to be subclinical or asymptomatic (infection is implied by a positive delayed-type hypersensitivity response to *Leishmania* antigens; DTH^+^-) [3]. When infection progresses to disease, it causes enlargement of spleen and liver and can cause hematological disorders, especially anemia, thrombocytopenia, which may cause bleeding, and neutropenia, resulting in increased host susceptibility to bacterial infection. In addition, patients with VL suffer weight loss and fever [3]. These complications may lead to the death if proper treatment is not provided. A recent study proposed a prognostic scoring system for VL patients, considered the following as predictors/ risks for death,: mucosal bleeding, dyspnea, jaundice, suspected or confirmed bacterial infection, neutropenia and thrombocytopenia [4].

The immune response of asymptomatic subjects is characterized by a T cell response against leishmania antigens displayed by positive DTH skin test [3],[5]. In contrast, classical VL patients present an impairment of IL-2, IFN-γ and IL-12 production from T cells specific to leishmania antigens *in vitro*, that is restored after successful treatment [6–14]. Interestingly, patients with active VL have high levels of IFN-γ and IL-12 in their serum, which theoretically could help to control the infection. Nevertheless, there is also an elevation of anti-inflammatory mediators, such as IL-10, that can encourage parasite multiplication, and interfere with the control of infection [10,15]. Several studies have described other serum cytokines as modulators of the immune response in VL patients, including IL-6 [7], IL-21 and IL-27 [16,17]. It is not currently known how closely levels of these cytokines correlate with the clinical outcome of VL nor is it known how these molecules are induced. Although the inflammatory response can be induced by the parasite alone, it can also be influenced by coinfections, especially with bacteria.

Patients with VL often present bacterial infection [9,18]. Sepsis caused by bacteria infection is characterized by the elevation of serum levels of lipolysaccharide (LPS) and sCD14 as a consequence of bacterial spread to the blood [19]. Santos-Oliveira et al. suggested the interference of bacterial co-infections in the severity of VL, based on the finding of high serum levels of LPS, sCD14, MIF and Fatty Acid-Binding Protein 2 (FABP2) [20]. This suggestion was based on observation of a few patients, however, and this interesting finding cannot yet be generalized as an explanation for disease severity in all VL patients.

The identification of biomarkers that can assess the severity of VL would provide insight toward disease pathogenesis and may contribute to improved clinical monitoring of patients. In this study, we observed high circulating levels of IFN-γ, IL-10, IL-6, TNF-α and IL-27 during active VL disease. We also determined the levels of some pattern recognition molecules, finding that although LPS levels were not different between healthy individuals and VL patients, circulating sCD14 was elevated during active VL. These data suggest that sCD14 could directly participate in modulating the immune response against *L*. *infantum*.

## Methods

### Study design and subjects

A cross-sectional study was performed between June 2011 and June 2013 in the VL Reference Center, University Hospital in Sergipe State, Brazil. A total of 65 subjects, including patients and household contacts, were enrolled. The clinical criteria used for inclusion were clinical symptoms and signs, such as fever, weight loss, anemia, enlargement of spleen and liver, pancytopenia and hypergammaglobulinemia. VL diagnosis was confirmed by direct observation of *Leishmania* in bone marrow aspirates or positive culture in NNN media (Sigma-Aldrich, St. Louis, MO), or positive rK39 serological test (KalazarDetect Rapid Test: InBios International Inc., Seattle, WA). Patients were submitted to standard VL treatment [21]. Pregnant women, patients receiving immunosuppressive treatments, and patients with comorbities such as diabetes, HIV, HTLV-1 and malignancy, were excluded. VL household contacts with a positive DTH skin test (Montenegro Skin test—Centro de Produção e Pesquisa de Imunobiológicos, Piraquara PR, Brazil) superior to 5 mm induration size [22], but without symptoms or signs of classical VL, were considered asymptomatic.

Information relating to demographic, clinical and laboratory features were collected following a standard protocol. The subjects were distributed in four groups: a) household contacts of VL patients with positive DTH skin test without symptoms or signs of classical VL, DTH^+^ (n = 11); b) patients with classical manifestation of VL before treatment, VL D0 (n = 25); c) patients with classical VL, 30 days after treatment, VL D30 (n = 17); d) patients classified as severe VL based on clinical features that included platelet counts <50,000/mm^3^, bleeding, bacterial infections, neutrophil counts <500/mm^3^, dyspnea and jaundice as described by Sampaio et al., [4], SVL (n = 12). A group of individuals whose residences were outside the *Leishmania*-endemic areas, and were not infected with other infectious disease, were also included and considered as healthy controls (HC) (n = 7).

### Detection of cytokines

Blood was collected from all subjects, serum prepared within 30 minutes and frozen at -80°C until use. The cytokine content of each sample was measured by multiplex assay, according to manufacturer’s instructions (Human Th17 Kit, Merck Millipore, Massachusetts, USA). The cytokines analyzed were IL-1β, IL-13, IL-12p70, IL-23, IL-17α, IL-4, IL-23, IFN-γ, IL-6, IL-10, IL-27 and TNF-α. The cytokines concentrations were analyzed by MILLIPLEX Analist 5.1 software (Merck Millipore, Billerica, USA).

### Measurement of LPS, sCD14, MIF and FABP2

The serum levels of the sCD14 (soluble Cluster Differentiation 14), MIF (Macrophage Migration Inhibitory Factor), FABP2 (Fatty Acid-Binding Protein 2 and LPS (lipopolysaccharide) were determined by ELISA, according to manufacturer’s instruction. Kits for detection of sCD14 (Quantikine; R&D Systems, Minneapolis, MN, USA) present a sensitivity of 125 pg\ml; MIF (Quantikine; R&D Systems, Minneapolis, MN, USA) detect a range 0.156–10 ng\ml; FABP2 (Duo set; R&D Systems, Minneapolis, MN, USA) present a sensitivity of 31.2 pg\ml; LPS (Limulus amebocyte lysate QCL-1000; Cambrex, Milan, Italy) present a sensitivity of 10 pg\ml, and the assay was performed twice.

### Statistical analysis

Descriptive and statistic data analysis were performed. D`Agostino and Pearson normality test was applied. Comparison between two groups was performed by Mann-Whitney Test. Spearman correlation test was used to correlate clinical and laboratorial data with the serum levels of immune molecules. All tests were carried out using Graph Pad Prism, version 4.0, 2005. Tests were considered statistically significant if the probability of a type I error was less than 5% (p value <0.05).

### Ethical considerations

This study was approved by the Ethics Committee of the Hospital Universitário of the Universidade Federal de Sergipe, Comissão Nacional de Ética em pesquisa (CONEP) CAAE 0151.0.107.000–07. All subjects, or their legal guardians, signed an informed consent form prior to the start of the study. Patients were treated with N-Methylglucamine antimoaniate, amphotericin B or liposomal amphotericin B according to the conventional protocol of the Brazilian Ministry of Health.

## Results

### Demographic, clinical and laboratorial data of patients

All patients had the diagnosis of VL confirmed by rK39 serology and/or bone marrow culture of parasites. About half of the subjects were females in each group, with the exception of the SVL group that was predominantly comprised of men (Table 1). The mean age of the VL D0 group was lower than the others, but this was not significantly different from the SVL group (31 ± 25 vs 15 ± 13, respectively; *p* = 0.13). No differences in spleen or liver sizes were detected between the VL D0 and SVL groups. The groups with active disease presented hematologic disorders, especially a decrease in platelet and neutrophil counts, and a greater drop was observed in the SVL group (Platelets 41,359/mm^3^ ± 66,401 and neutrophils 423.4/mm^3^ ± 324.0) as compared to the VL D0 group (Platelets 121,130/mm^3^ ± 80,496 and neutrophils 1,106/mm^3^ ± 820.5) (p≤0.005 and p≤0.05, respectively). After treatment, the patients recovered, or showed signs of recovery of, these hematologic parameters. Similarly, hepatic enzymes alanine aminotransferase (ALT) and aspartate aminotransferase (AST) were elevated in groups with active disease but had resolved to normal range after treatment. The asymptomatic (DTH^+^) individuals did not present clinical or laboratorial abnormalities.

**Table 1:** Demographic and clinical characteristics of the studied subjects.

	Healthy Control—HC (n = 7)	DTH^+^ (n = 11)	Visceral Leishmaniasis pre-treatment—VL D0 (n = 25)	Visceral Leishmaniasis pos-treatment—VL D30 (n = 17)	Severe Visceral Leishmaniasis–SVL (n = 12)
Gender (M/F)	1/6	5/6	12/13	9/8	11/1
Mean age (min/max)	33(24/50)	25(3/50)	15(0.7/44)	16(0.7/44)	31 (1/65)
**Clinical dat**a (**mean ± SD)**					
Spleen Enlargement (cm)	0 ± 0	0±0	9.5±5.6	3.3±3.6^c^	10.6±4.1
Liver Enlargement (cm)	0 ± 0	0.5±1.5	5.0±3.2	2.1±1.9^c^	6.2±3.8
**Hematological data** (**mean ± SD)**					
Hematocrit (%)	-	40.2±4.0	25.1±4.1	32.7±3.7^c^	23.8±4.8
Hemoglobin (g/dL)	-	13.2±1.4	7.9±1.3	10.6±1.3^c^	7.6±1.5
Platelet (/mm^3^)	-	240,273±5,145	121,130±80,496	245,125±72,475^c^	41,359±66.40^b^
Neutrophil (/mm^3^)	-	2,873±1,006	1,106±820.5	2,358±875.2^c^	423.4±324.0^a^
Eosinophils (/mm^3^)	-	645.9±538.8	71.4±173.6	344.9±367.2^c^	17.0±33.5
**Hepatic enzymes** (**mean ± SD)**					
AST (U/L)	-	24.6±12.0	101.4±69.49	44.1±23.0^b^	44.1±36.8^b^
ALT (U/L)	-	15.6±6.8	56.9±38.2	38.1±34.9^a^	53.0±146.8^c^
γGT (U/L)	-	19.7±7.9	85.7±71.5*	48.1±35.2**	123.3±61.1
Alkaline phosphatase (U/L)	-	137.6±91.4	206.4±216.5*	111.3±99.1**	228.9±125.8

a–*p* ≤ 0.05

b–*p* ≤ 0.005

c–*p* ≤ 0.0005 (VL D0 comparation)

*n = 10

**n = 8

### Circulating cytokine profile in VL patients

**Fig 1:**
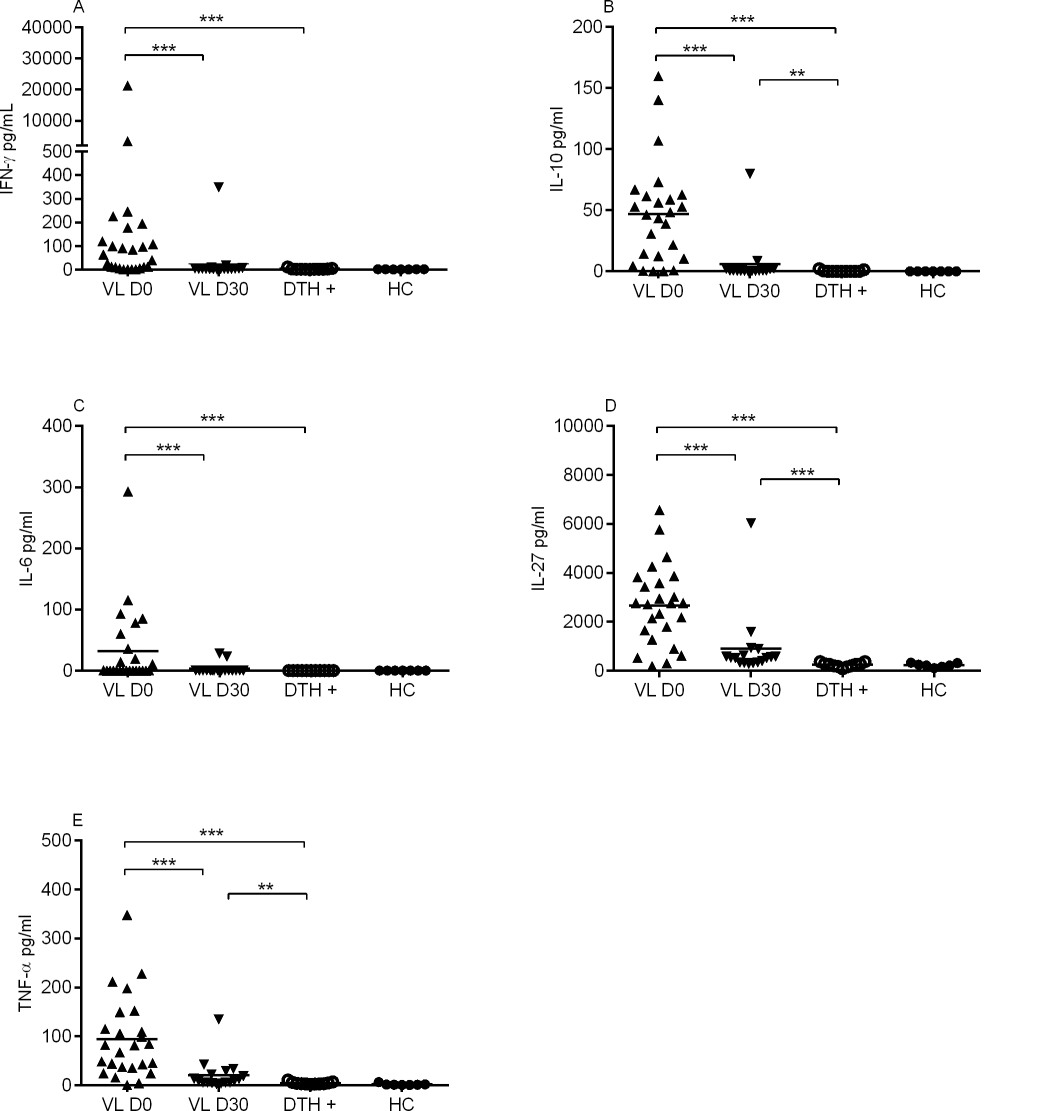
Serum levels of cytokines. Cytokines were measured by Luminex assay in sera of VL patients before (n = 25) and after treatment (n = 17) (D0 and D30, respectively), DTH+ (n = 11) and healthy control (n = 7). (A) IFN-γ (B) IL-10, (C) IL-6, (D) IL-27 and (E) TNF-α.

High levels of IFN-γ, IL-10, IL-6, IL-27 and TNF-α cytokines were observed in sera of VL patients before treatment when compared with healthy control and DTH+ individuals (Fig 1). Additionally, the serum levels of these cytokines decreased significantly after treatment: IFN-γ (1,053 ± 4,267 pg/ml to 24 ± 83 pg/ml), IL-10 (46 ± 41 pg/ml to 5 ± 19 pg/ml), IL-6 (32 ± 64 pg/ml to 3 ± 8 pg/ml), IL-27 (2,675 ± 1,630 pg/ml to 895 ± 1,361 pg/ml) and TNF-α (94 ± 82 pg/ml to 20 ± 31 pg/ml) (VL D0; Fig 1).

### Cytokines levels correlate with clinical, hematological and biochemical parameters

We then evaluated how cytokine levels related to patients’ clinical profile (Table 2). Spleen and liver enlargement, the classical manifestation of VL, both strongly and positively correlated with the circulating levels of IL-10, IL-6, IL-27 and TNF-α (all *p*≤0.0005). Similarly, serum levels of ALT and AST, which are also considered as markers of disease severity, positively correlated with TNF-α, IL-27 and IL-10 (*p* ≤0.0005).

**Table 2:** Correlation between serum cytokine levels, and clinical and hematological data of the all patients.

Clinical and laboratorial data	Biomolecules				
	TNF-α	IFN-γ	IL-6	IL-10	IL-27
**Clinical data**					
Spleen size (cm)	0.64^c^	0.37^b^	0.52^c^	0.56^c^	0.65^c^
Liver size (cm)	0.63^c^	0.45^c^	0.50^c^	0.57^c^	0.72^c^
**Hematological data**					
Hemoglobin (g/dL)	-0.69^c^	-0.46^c^	-0.66^c^	-0.60^c^	-0.65^c^
Platelet (/mm^3^)	-0.61^c^	-0.44^c^	-0.53^c^	-0.53^c^	-0.62^c^
Neutrophil (/mm^3^)	-0.60^c^	-0.33^b^	-0.54^c^	-0.46^c^	-0.64^c^
Eosinophils (/mm^3^)	-0.56^c^	-0.49^c^	-0.53^c^	-0.54^c^	-0.59^c^
**Hepatic enzymes**					
AST (U/L)	0.49^c^	0.34^b^	0.26^a^	0.45^c^	0.45^c^
ALT (U/L)	0.30^a^	0.34^b^	0.00	0.36^b^	0.32^a^

All numbers represent the Spearman r.

^a^ *p* ≤ 0.05

^b^ *p* ≤ 0.005

^c^ *p* ≤ 0.0005

Hematological disorders, such as hemoglobin concentration, negatively correlated with TNF-α, IL-6, IL-27 and IL-10 (all *p* ≤0.0005); Platelet and eosinophil counts were negatively correlated with TNF-α, IL-6, IL-27 and IL-10 (*p* ≤0.0005). Neutrophil count negatively correlated with TNF-α, IL-27 and IL-6 (*p* ≤0.0005).

### Correlation between cytokine levels and disease severity

**Fig 2:**
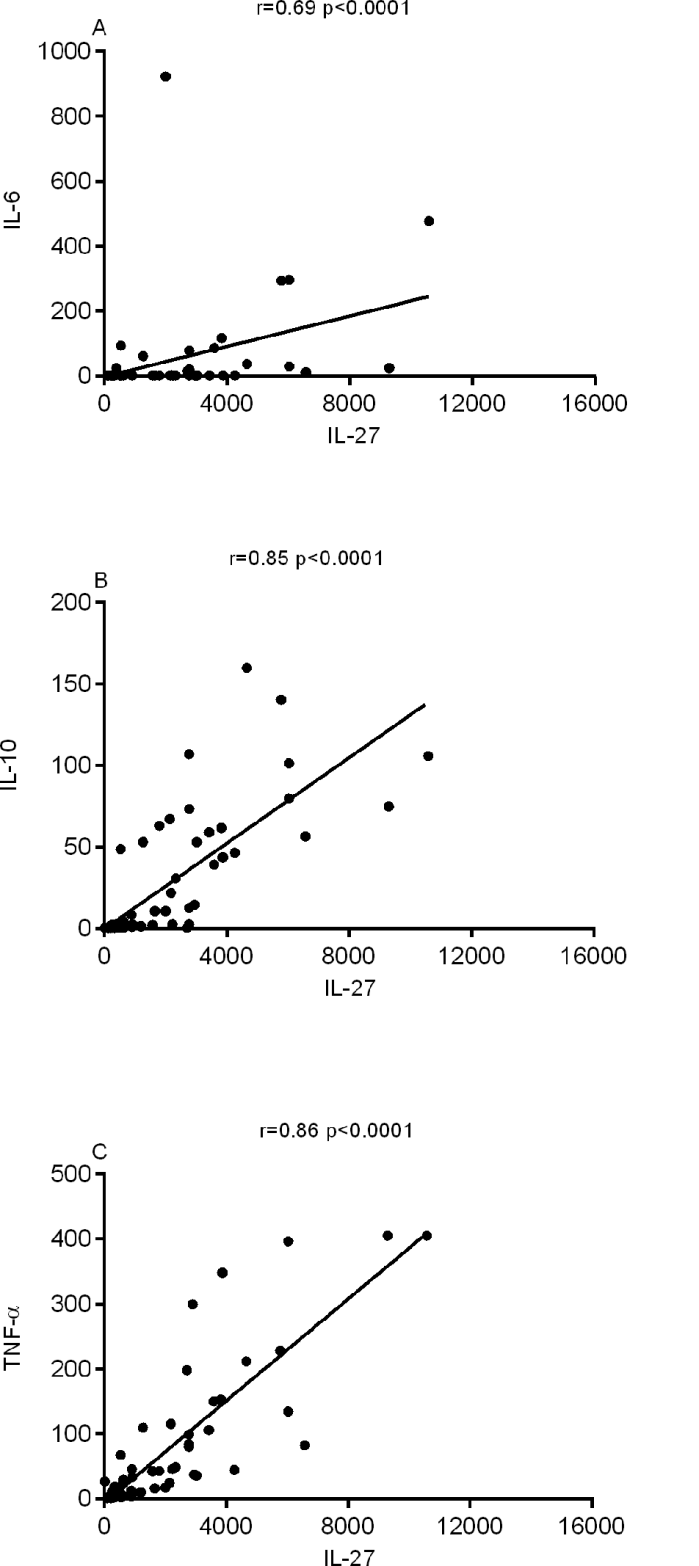
Correlation between the cytokines levels. Serum levels of IL-27, IL-6, IL-10 and TNF-α were measured in VL patients before treatment by Luminex assay. Correlation was observed between IL-27 with the cytokines IL-6 (A), IL-10 (B), TNF-α (C). Spearman correlation test.

The levels of particular circulating cytokines during the inflammatory processes observed in active VL disease could be a determinant, or arise as a consequence, of disease severity. Thus, we evaluated the relationship of molecules typically associated with the cytokine storm phenomenon. We observed a strong correlation between IL-27 and IL-6 (r = 0.69), IL-10 (r = 0.85) and TNF-α (r = 0.86) (Fig 2).

**Fig 3:**
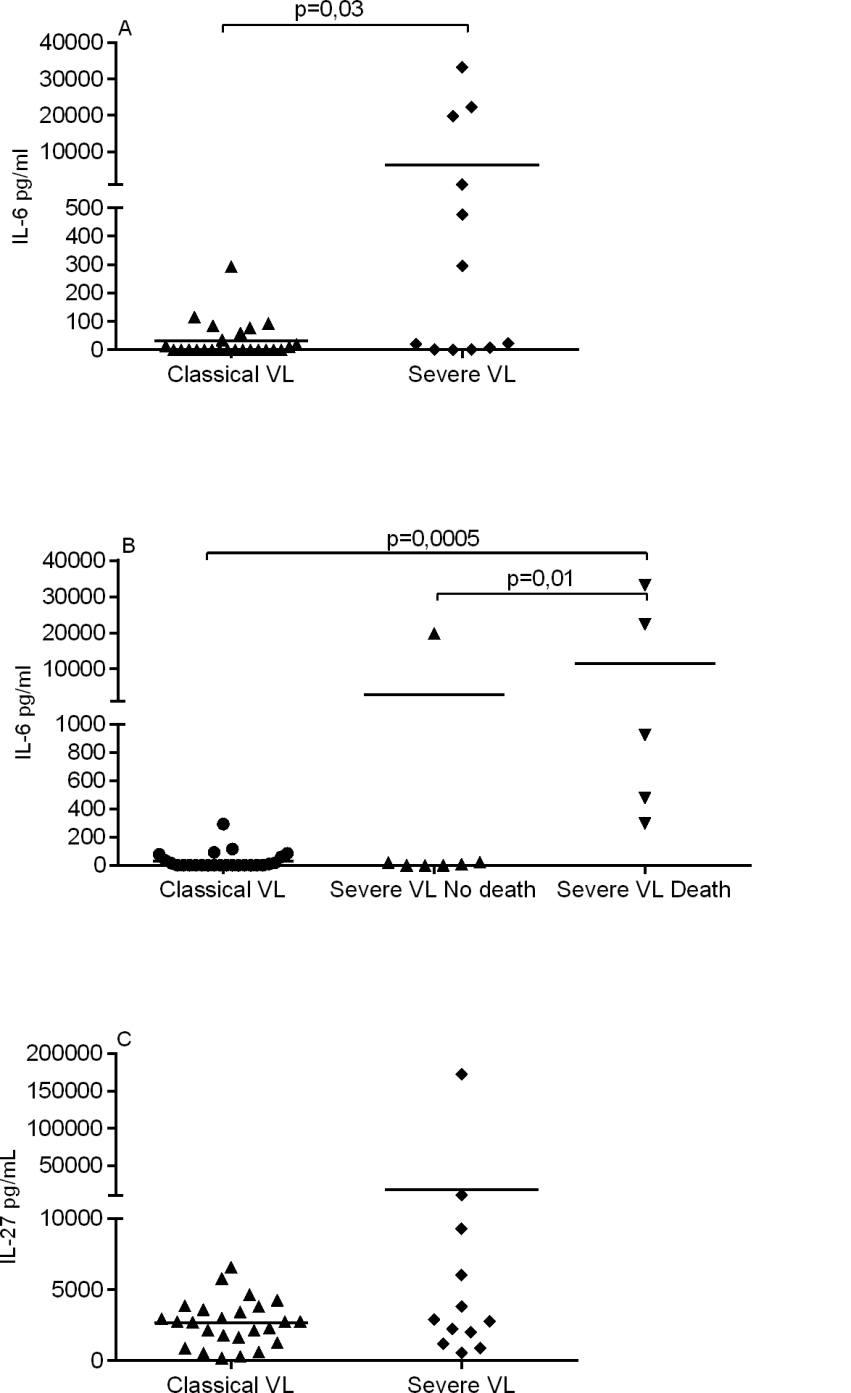
High IL-6 levels in serum is associated with disease severity. Serum levels of IL-6 and IL-27 measured before treatment by Luminex assay were compared in VL patients. (A) IL-6 levels in patients with classical VL (n = 25) and SVL (n = 12). (B) IL-6 levels in patients with classical VL (n = 25) and SVL that survived (n = 7) and SVL that died (n = 5). (C) IL-27 levels in patients with classical VL (n = 25) and SVL (n = 12).

Further stratification of the cytokine profiles to allow comparison between classical VL and severe VL presentations revealed that IL-6 levels were more significantly elevated in the patients with severe VL (Fig 3A; *p* = 0.03). Remarkably, severe VL patients that died had IL-6 serum levels of 11,443 ± 15,388 pg/ml, whereas the surviving severe VL patients had 2,848 ± 7,512 pg/ml (Fig 3B; *p* = 0.01). All of the patients that died had circulating IL-6 levels ≥ 200 pg/ml, while only 2 patients that survived (1 SVL, 1 classical VL) presented with IL-6 levels above this threshold. Although not statistically significant, SVL patients presented higher levels of IL-27 compared with classic VL (17358 ± 48765 pg/ml versus 2675 ± 1630 pg/ml) (Fig 3C). Significant differences were not observed between VL D0 and SVL groups for the other cytokines examined.

### Pathogen recognition molecules and VL

**Fig 4:**
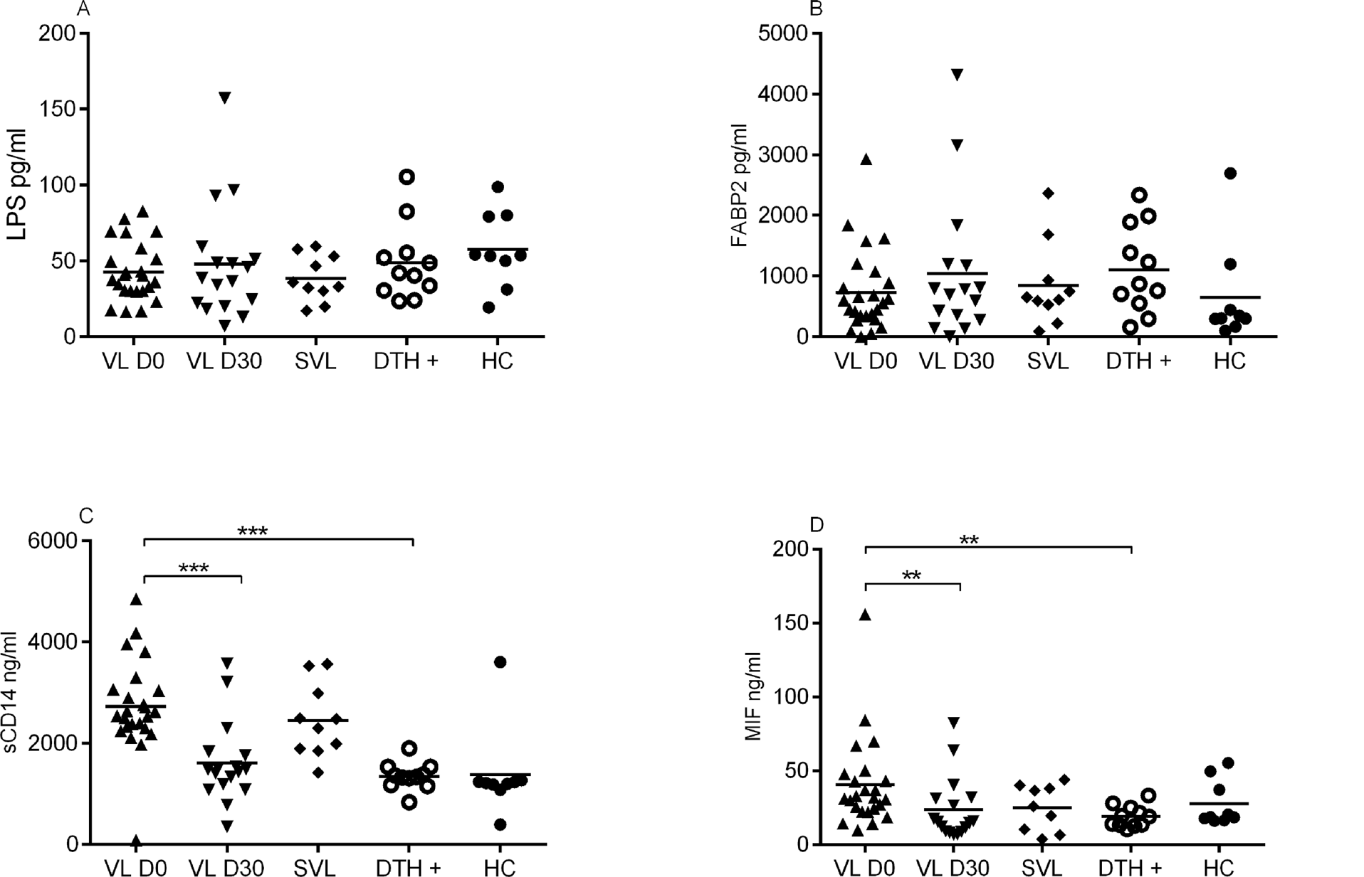
Levels of sCD14 and MIF are elevated in the serum of VL patients. Serum levels of the LPS (A), FABP2 (B), sCD14 (C) and MIF (D) were measured by ELISA in classical VL patients before (n = 25) and after treatment (n = 17) (D0 and D30, respectively), severe VL patients (n = 12), DTH+ (n = 11) and healthy control (n = 07). * Mann-Whitey test.

Recent reports have indicated a link between bacterial co-infection and VL disease severity [20,23,24]. To examine if bacterial infections could be influencing the release of the inflammatory and anti-inflammatory cytokines, we measured levels of the LPS, FABP2, MIF and sCD14 molecules. LPS and FABP2 levels were similar in the VL D0, DTH+ and HC groups and were not changed after treatment (VL D30) (Fig 4A and 4B). However, sCD14 and MIF, molecules associated with TLR recognition, were higher in the active disease group (2,882 ng/ml and 39 ng/ml, respectively) than DTH+ group (1,348 ng/ml and 19 ng/ml, respectively). Furthermore, circulating levels of sCD14 and MIF decreased with treatment (1,820 ng/ml and 17 ng/ml, respectively) (Fig 4C and 4D).

MIF showed a low correlation with both clinical and laboratorial parameters (S1 Table). The sCD14 concentrations, however, correlated with spleen and liver size, hematocrits, hemoglobin concentrations, and number of the neutrophils, platelets and eosinophils (Table 3). sCD14 levels also correlated with IL-10, IL-6, TNF-α and IL-27 (Table 3). sCD14 levels were not, however, associated with disease severity/ mortality.

**Table 3:** Correlation coefficients of sCD14 levels and clinical, hematological data and cytokine concentrations of the all patients.

Clinical and laboratorial evolution	sCD14	
	r	p
Spleen size (cm)	0.67	< 0.0001
Liver size (cm)	0.52	< 0.0001
Hemoglobin (g/dL)	-0.64	< 0.0001
Platelet (/mm^3^)	-0.51	< 0.0001
Neutrophil (/mm^3^)	-0.53	< 0.0001
Eosinophils (/mm^3^)	-0.53	< 0.0001
AST (U/L)	0.55	< 0.0001
ALT (U/L)	0.22	0.0687
TNF-α	0.49	< 0.0001
INF-γ	0.35	0.0042
IL-6	0.55	< 0.0001
IL-10	0.55	< 0.0001
IL-27	0.47	0.0001

## Discussion

In the present study we not only confirm the presence of high serum levels of inflammatory and anti-inflammatory cytokines in active VL, but describe for the first time high concentrations of IL-27 and its correlation with clinical profile, whilst additionally showing that the presence of sCD14 in sera is not associated with bacterial infection.

We found gender and age to be associated with disease outcome and a higher number of males presented in the SVL group. Herein, most of VL patients with the classical VL were younger than 15 years old, while the SVL patients were older than 40. In a meta-analysis study, Belo et al. [25] showed that children are more susceptible to developing VL, and severe disease and death occurs more frequently in individuals under 1 year or older than 50 years old [26,27]. In our SVL group, of the five patients that died, two were younger than 5 years while three were older than 49 years old. Differences in age could also partly explain the gender observation, as it has been reported that in Brazil subjects older than 10 years the ratio of male:female ratio for VL tends to be much higher [23]. This profile could arise due to increased exposure to sandflies and greater parasite loads [28], or due to the effects of testosterone which has been associated with both lesion size in infected hamsters and an increase of *L*. *donovani* replication in infected macrophages [29,30].

Systemic inflammation has been described as a cause of clinical manifestations in several intracellular infections, including malaria [31] and leishmaniasis [32–35]. In VL, this inflammatory response presents as a release of multiple cytokines, well described in the literature as cytokine storm [6–9,13,14]. The cytokine storm could be considered as the direct cause of some clinical manifestations of VL. Indeed, the release of inflammatory mediators could be responsible for tissue damage, and their influence on the severity of VL was previously indicated by Costa et al 2010, 2013 [7,34]. Our data therefore support those earlier observations. The evidence that the systemic release of cytokines favors evolution/severity of the disease is supported by the observation that asymptomatic individuals do not present with such a profile [6–9,13,14]. Presumably, while the parasites may directly harm the host, they may also indirectly cause damage through the host response. Our data reinforce the potential clinical impact of this by correlating cytokine levels (especially IFN-γ, IL-10, IL-6, IL-27 and TNF-α) with clinical manifestations of VL associated with disease severity, such as spleen and liver size, lower neutrophil and platelets counts, and greater release of liver enzymes. Although IFN-γ is considered to be involved in parasite control, its ability to exert this function appears to be suppressed by down modulatory cytokines also released in sera, such as IL-10 [5,36,37]. It is still unclear why IFN-γ does not overcome the down modulatory effect of IL-10, but it is possible that a specific activation mediated by T cell signaling is needed to more efficiently activate the macrophage microbicidal mechanisms. The absence of specific T cell responses, that are recovered after treatment, is well described for VL [5,38]. It is also well known that the interaction of T cells and macrophages involves a complex cell signaling pathway including engagement of MHC-peptide-TCR and co-stimulatory molecules, such as CD40-CD40L, in addition to cytokine release [39]. The negative correlation between levels of soluble CD40 ligand, parasite load and spleen size observed in *L*. *infantum* infection suggests that cell signaling might help to control the disease [40]. The nonspecific secretion of IFN-γ has been associated with complications in other inflammatory diseases [41,42]. However, the present study does not indicate significant differences in IFN-γ levels between classical (VL D0) and severe (SVL) VL patients.

Conversely, it is possible that the timing of release of each cytokine orchestrates an immunosuppressive profile that influences disease outcome. For instance, high levels of IL-10 were present in sera of VL but absent in asymptomatic individuals. IL-10 can protect tissues from colateral damage caused by excessive inflammation [43], but in infectious diseases, where an inflammatory response is required to prevent parasite proliferation, IL-10 may prevent an ideal response [36]. Several studies have associated the presence of IL-10 in the serum, PBMCs, spleen, liver or lymph nodes of VL patients with disease severity (reviewed in [15]) and previous studies reported an inverse correlation of IL-10 levels with parasite loads in bone marrow [44] and the blood [8]. Although they did not measure IL-10, Silva et al., 2014 correlated parasite load with disease severity [45]. Costa et al., 2013 found an association of IL-10 with some clinical indicators of disease severity, such as bleeding, neutrophil counts and hemoglobin levels [7]. Furthermore, neutralization of IL-10 in splenic aspirate cells from VL patients increases IFN-γ and TNF-α production, and reduces parasite burden [10].

We observed strong correlations between IL-27, IL-10, IL-6 and TNF-α. IL-27 has been demonstrated to induce Th1, Th2 or Treg responses (reviewed in [17,46], but in VL, IL-27 is a disease promoter, stimulating the *in vitro* differentiation and expansion of IL-10 producing T cells [16,46]. This ability to increase IL-10 can be triggered by different pathways [17]. IL-27 also promotes B cell activation, aggravating the hypergammaglobulinemia observed in VL patients [17]. Our data correlate IL-27 with IL-6. We also found that high levels of IL-6 were associated with lethality, confirming the earlier report by Costa et al., [7]. IL-6 can directly inhibit TNF-α production [47] and also suppress Th1 responses [48] or induce Th2 responses [49]. Thus, the strong association of IL-6 with disease severity and death might be explained in two steps: firstly, through the inhibition of TNF-α in the early phase of infection, and later, by its inhibitory effect on Th1 responses [7].

During active disease, however, TNF-α is also high in VL patients and is positively correlated with IL-27. The well known proinflammatory effect of TNF-α, particularly in synergising with IFN-γ, could activate macrophages for clearance of the parasite. Another possible explanation was described by Peruhype-Magalhães et al. [14], who documented high levels of the TNF-α in the serum of active VL patients despite low circulating numbers of TNF-α^+^ monocytes. The TNF-α released in serum can be responsible for the clinical manifestation of the disease, as demonstrated by direct correlation with several clinical markers of disease severity. The deleterious effect of the systemic release of TNF-α has been described in several diseases [50–52].

VL patients also frequently present with bacterial infections [9,18] and Santos-Oliveira et al, 2010 associated this release of the inflammatory and anti-inflammatory mediators with bacterial co-infection [20]. In an attempt to clarify if the release of the inflammatory and anti-inflammatory mediators was associated with bacterial co-infection, as suggested by Santos-Oliveira et al. [20], we examined our samples for the presence of LPS, MIF, FABP2 and sCD14. Although bacterial infections are observed in patients with VL [9,18] our data, generated with a higher number of patients than previously evaluated, appear to rule out the influence of bacterial infections. Disease was not associated with either high serum levels of the LPS or FABP2. However, sCD14 and MIF were high during disease and decreased after treatment. Furthermore, the levels of sCD14 correlated with clinical and laboratory manifestations of VL, and also with IL-10, IL-6, IL-27 and TNF-α levels. These data indicate that parasite antigens trigger CD14. CD14 is a membrane glycoprotein on monocytes and macrophages that acts as co-receptor to facilitate the recognition of LPS in conjunction with TLR4 [20,53]. TLR4 and TLR2 is expressed in lesions of cutaneous leishmaniasis and TLR2 in blood cells of VL patients, suggesting that these signaling molecules are involved in *Leishmania* infections [54]. CD14 can also be soluble (sCD14), and as it is able to activate endothelial cells, the soluble form can assist in the recognition of circulating pathogens [55,56]. Furthermore, sCD14 can inhibit the macrophage activation and decrease the inflammatory response [57]. The activation of CD14 during leishmania infection could trigger the release of inflammatory mediators by macrophages and other cells, in association with either TLR2 and TLR4.

Considering our data together, we hypothesize that *Leishmania* may trigger sCD14 and start an inflammatory response by stimulating IL-27, IL-10 and IL-6 production. These cytokines down regulate macrophage microbicidal activity, facilitating leishmania proliferation. Release of IL-6 in the early stages of the infection might interfere with the induction of a specific Th1 response, predisposing the parasites to proliferate and cause disease [47]. High IL-6 levels are associated with the most severe cases and death. As infection progresses, the parasite might trigger CD14 and other signaling molecules, inducing the release of both anti-inflammatory and pro-inflammatory molecules, with a persistent absence of specific IFN-γ response being the halmark of classical VL. Further follow-up and *in vitro* studies are needed to test these hypotheses and clarify the sequential release of the immune mediators, as well as to understand the mechanisms involving CD14 and its influence in the immunopathogenesis of VL.

## Supporting Information

S1 Table MIF correlations. (DOCX)
