# Supplementary material for: The Severity of Visceral Leishmaniasis Correlates with Elevated Levels of Serum IL-6, IL-27 and sCD14
Source: PLoS Negl Trop Dis. 2016 Jan 27;10(1):e0004375. doi: 10.1371/journal.pntd.0004375 (PMC4729473; doi:10.1371/journal.pntd.0004375)
Supplement: S1 Table — (DOCX) [file pntd.0004375.s001.docx]

Suplementary Table 1: MIF correlations.

| **Clinical and laboratorial evolution** | **MIF** | |
| --- | --- | --- |
|  | **r** | **p** |
| Spleen size (cm) | 0.2057 | 0.0697 |
| Liver size (cm) | 0.1283 | 0.1823 |
| Hematocrit (%) | -0.3231 | 0.0134 |
| Hemoglobin (g/dL) | -0.6423 | < 0.0001 |
| Platelet (/mm^3^) | -0.2769 | 0.0298 |
| Neutrophil (/mm^3^) | -0.1678 | 0.1297 |
| Eosinophils (/mm^3^) | -0.1678 | 0.1297 |
| AST (U/L) | -0.2771 | 0.0297 |
| ALT (U/L) | 0.3702 | 0.0062 |
| γGT (U/L) | 0.2902 | 0.0266 |
